# Benchmarking of radiobiological NTCP models in head and neck radiotherapy using independent computational pipelines: an institutional validation study with machine learning augmentation

**DOI:** 10.3332/ecancer.2026.2147

**Published:** 2026-06-16

**Authors:** Kalyan Mondal, Abhijit Mandal, Anuj Vijay, Ganeshkumar Patel

**Affiliations:** 1 Department of Physics, Institute of Applied Science & Humanities, GLA University, Mathura, Uttar Pradesh 281406, India; 2 North Bengal Medical College, Sushrutanagar, Darjeeling 734012, India; 3 Department of Radiotherapy and Radiation Medicine, Institute of Medical Sciences, Banaras Hindu University, Varanasi, Uttar Pradesh 221005, India; a http://orcid.org/0000-0001-7685-7391; b http://orcid.org/0000-0001-5626-1072; c http://orcid.org/0000-0001-6610-3844; d http://orcid.org/0000-0002-1876-6069

**Keywords:** normal tissue complication probability, radiobiological models, dose-volume histogram, machine learning, head and neck neoplasms

## Abstract

**Background & purpose:**

Normal tissue complication probability (NTCP) models require institutional validation before clinical implementation. Traditional radiobiological models, such as the Lyman–Kutcher–Burman (LKB) and Equivalent Uniform Dose (EUD) models, provide mechanistic dose–response frameworks, while machine learning (ML) approaches offer exploratory, data-driven alternatives that remain inadequately characterised in South Asian populations.

**Methods:**

This retrospective study included 51 head and neck cancer patients treated with definitive radiotherapy. Binary endpoints were Grade ≥2 xerostomia (n = 3), dysphagia (n = 5) and mucositis (n = 4), scored using Common Terminology Criteria for Adverse Events version 5.0. NTCP calculations were performed using two independent computational pipelines (MATLAB-based RBMODELv1 and a Python implementation), with agreement assessed using Bland–Altman analysis. Traditional NTCP models (LKB, EUD) were evaluated and compared with artificial neural networks and XGBoost in a hypothesis-generating framework using a stratified 70:30 train–test split. Model performance was assessed using the area under the receiver operating characteristic curve (area under the curve), accuracy and Spearman’s rank correlation.

**Results:**

Excellent agreement was observed between computational pipelines (mean bias 0.8%, 95% limits −1.9% to 3.5%). Traditional models demonstrated strong rank-order correlation with toxicity grades (ρ = 0.61–0.79, p < 0.001) and high accuracy (LKB: 90.0%–94.1%). Institution-specific parameters differed from quantitative analyses of normal tissue effects in the clinic values, including a lower parotid TD50 (34.1 versus 39.0 Gy). Exploratory ML analyses showed numerically higher discrimination for parallel organs but not for mixed-architecture structures; however, severe class imbalance (3–5 events per endpoint) limits statistical reliability.

**Conclusion:**

Dual computational pipelines enable reproducible NTCP modeling for institutional use. Traditional radiobiological models perform acceptably after local calibration, while exploratory ML findings suggest potential organ-architecture-dependent patterns that require validation in adequately powered multi-institutional cohorts.

## Introduction

Head and neck cancer radiotherapy achieves high locoregional control rates exceeding 70% for locally advanced disease, but radiation-induced toxicities, including xerostomia, dysphagia and mucositis, significantly impact quality of life and functional outcomes [1–3]. Modern treatment planning relies predominantly on empirical dose-volume histogram (DVH) constraints that provide binary pass or fail assessments without quantifying actual complication risks [4]. Normal tissue complication probability (NTCP) models offer continuous risk estimates by integrating dose-volume data into mechanistic frameworks, enabling more nuanced treatment plan evaluation and optimisation [5–7].

The Lyman-Kutcher-Burman (LKB) model remains the most widely validated NTCP framework, employing a cumulative normal distribution with three tissue-specific parameters, TD50 (dose causing a 50% complication probability), m (slope of the dose-response curve) and n (volume effect parameter) [8, 9]. The Equivalent Uniform Dose (EUD) formalism reduces heterogeneous dose distributions to biologically effective uniform doses through the generalised EUD (gEUD) concept, with organ-specific volume parameter ‘a’, reflecting architectural tolerance patterns [10, 11]. The Relative Seriality (RS) model explicitly incorporates organ functional subunit architecture, distinguishing parallel organs (functional redundancy) from serial organs (sequential vulnerability) through the seriality parameter ‘s’ [12, 13]. These models underpin the Quantitative Analyses of Normal Tissue Effects in the Clinic (QUANTEC) guidelines that established dose-volume constraints based on pooled literature analysis [14–17].

Despite extensive validation in Western populations, [15–17] several critical knowledge gaps remain. First, QUANTEC parameters were derived predominantly from European and North American cohorts, with limited validation in Asian populations, where genetic polymorphisms [18–20], lifestyle factors [21–23] and treatment protocols [24–27] may influence toxicity profiles. Second, computational implementation varies across software platforms, potentially affecting prediction consistency and clinical adoption [28–30]. Third, while machine learning (ML) approaches demonstrate promise in multi-factorial prediction [31–33], their performance relative to mechanistic models remains incompletely characterised, particularly across organs with distinct architectural properties [34]. Parallel organs such as parotid glands exhibit volume-dependent tolerance through functional subunit redundancy, whereas serial structures like pharyngeal constrictors demonstrate threshold-based dose-response patterns [12, 13]. Whether data-driven approaches offer advantages over mechanistic models in these distinct scenarios requires systematic investigation [34–36]. Furthermore, if population-specific factors alter toxicity risk, purely mechanistic models may be limited [37]. Data-driven ML approaches, capable of integrating dosimetric, clinical and potentially genomic variables, may offer a complementary path for personalised prediction in distinct cohorts [38]. Consequently, ML models are best viewed as exploratory, hypothesis-generating tools rather than replacements for mechanistic NTCP models at the current stage of evidence [38, 39].

This institutional validation study addresses these gaps through three primary objectives: (1) to validate traditional NTCP models (LKB, EUD and RS) using dual independent computational pipelines to ensure reproducible implementation; (2) to derive institution-specific model parameters reflecting local patient demographics, treatment protocols and toxicity assessment methods and (3) to conduct a hypothesis-generating exploratory comparison with contemporary ML algorithms (artificial neural networks (ANNs) and extreme gradient boosting) to explore potential organ-architecture-dependent performance patterns that can inform future multi-institutional validation. Within the constraints of an exploratory, single-institution cohort, this study establishes baseline NTCP model performance in an Indian population and identifies specific research questions requiring validation through multi-institutional collaboration.

## Materials and methods

### Study design and patient selection

This retrospective study was conducted at a tertiary cancer care institute in India between March 2024 and September 2024. A total of 63 consecutive patients with histologically confirmed head and neck squamous cell carcinoma treated with definitive radiotherapy were initially identified. Inclusion criteria comprised age ≥18 years, Karnofsky performance status ≥70, Stage I–IVB disease according to the American Joint Committee on Cancer 8th edition and completion of the prescribed radiotherapy course, with sufficient treatment planning data available to permit DVH analysis. Exclusion criteria included prior head and neck radiotherapy, distant metastases at diagnosis, loss to follow-up before the first toxicity assessment or incomplete DVH data precluding NTCP modeling. After applying these criteria, 12 patients were excluded (incomplete DVH data, *n* = 7; loss to follow-up, *n* = 3; baseline xerostomia before radiotherapy, *n* = 2), resulting in 51 analysable patients (81%).

### Sample size and statistical considerations

This study’s retrospective design and limited sample size constrain the statistical strength of our findings. A post-hoc power analysis revealed that, with 51 patients and observed event rates of 5.9%–9.8%, the study was underpowered (30%–40%) to detect area under the curve (AUC) differences of 0.15 between models at *α* = 0.05 [40]. Furthermore, adhering to the ‘events per variable’ (EPV) guideline of ≥10 events per predictor, [41, 42] our endpoints provided only 3–5 events. This restricts traditional modeling to a maximum of 1–2 univariate predictors and renders any multivariable ML model suitable only for exploratory, hypothesis-generating analysis. Therefore, all multivariable analyses, especially those employing ML, must be interpreted as hypothesis-generating. The sample size is sufficient to identify general performance trends and generate organ-specific hypotheses, but is inadequate for definitive conclusions on model superiority. We explicitly acknowledge these constraints and contextualise our findings accordingly [43]. This approach is consistent with prior single-institution NTCP benchmarking studies with comparable event rates.

### Treatment planning and delivery

Treatment plans were generated using the Eclipse Treatment Planning System (version 15.6; Varian Medical Systems, Palo Alto, CA, USA). Patients received either three-dimensional conformal radiotherapy (3DCRT, *n* = 23, 45%) or volumetric modulated arc therapy (VMAT, *n* = 28, 55%). The prescribed dose was 66–70 Gy in 2-Gy fractions (median 68 Gy) delivered over 6.5–7 weeks. Organs at risk were delineated following international consensus guidelines [44]. Concurrent chemotherapy (weekly cisplatin 40 mg/m² or cetuximab loading 400 mg/m² followed by weekly 250 mg/m²) was administered to 21 patients (41.2%) per institutional protocol.

### Toxicity assessment

Toxicity was assessed using Common Terminology Criteria for Adverse Events version 5.0 (CTCAE v5.0) at baseline, weekly during treatment, and at 3, 6, 9 and 12 months post-treatment. Toxicity grading was primarily based on patient-reported symptoms captured using validated EORTC QLQ-C30 and QLQ-H&N35 instruments, with CTCAE v5.0 grades assigned accordingly. The primary binary endpoint was Grade ≥2 toxicity, aligning with QUANTEC guidelines and most comparative literature. This threshold represents clinically meaningful impairment requiring intervention: Grade ≥2 xerostomia indicates moderate symptoms limiting self-care, Grade ≥2 dysphagia requires dietary modification and Grade ≥2 mucositis necessitates analgesic support.

### DVH extraction and processing

Cumulative DVHs were extracted from archived treatment plans using the Eclipse scripting interface. DVH files contained dose-volume data at 0.1-Gy resolution, exported in text format with two columns: absolute dose (cGy) and absolute volume (cm³). Analysed organs included bilateral parotid glands, larynx and oral cavity. For bilateral parotid analysis, the ipsilateral and contralateral glands were combined as recommended by QUANTEC [15].

### NTCP model implementations

NTCP calculations were performed using two independent computational pipelines to ensure reproducibility and implementation robustness.

*Pipeline 1 – RBMODELv1 (MATLAB R2021a):* A MATLAB-based NTCP calculation platform developed and previously validated at our institution [45]. The software incorporates Niemierko’s EUD formalism with documented corrections to published code implementations and supports LKB, gEUD and RS models.

*Pipeline 2 – Python Implementation (Python 3.9):* An independent custom implementation developed using NumPy (v1.21) and SciPy (v1.7), publicly available via a GitHub repository. All algorithms were implemented using identical mathematical formulations and numerical conventions to those used in RBMODELv1.

All NTCP formulations followed canonical published definitions for the LKB, gEUD and RS models [8–13]. The gEUD formalism provides a unifying framework, of which the LKB EUD and Niemierko EUD formulations represent specific parameterisations.

For the LKB model, NTCP was calculated using the Lyman probit formulation with the Kutcher–Burman dose–volume reduction method [9]:

NTCP_LKB_ = Φ(t)

where,

t = (EUD–TD50)/(m × TD50)

and the EUD for the LKB model was computed as follows:

EUD = (Σ_i_ v_i_ D_i_^(1/n)^)^n^.

Here, Φ(t) denotes the cumulative distribution function of the standard normal distribution, v_i_ represents the fractional volume receiving dose D_i_, TD50 is the dose associated with 50% complication probability for uniform irradiation, ‘m’ is the slope parameter and ‘n’ characterises the volume effect.

The gEUD formulation was expressed as follows [11]:

gEUD = (Σ_i_ v_i_ D_i_^a^)^(1/a)^

where the parameter ‘a’ characterises organ architecture, with large negative values corresponding to parallel organs and large positive values corresponding to serial organs.

The RS model was implemented according to published formulations [12]:

$${\mathrm{NTCP}}_{\mathrm{RS}}=1-{\prod_{i}[1-P(D_{i}{)}^{s}]}^{(v_{i}/s)}$$

where,

P(D_i_) = 1/[1 + (D50/D_i_)^k^]^.^

Here, P(D_i_) is the probability that a small fractional volume of an organ will develop a complication if it receives dose D_i_, ‘s’ denotes the RS parameter (s → 0 for parallel organs, s → 1 for serial organs), D50 represents the dose associated with 50% complication probability for uniform whole-organ irradiation and ‘k’ defines the slope of the dose–response relationship.

Initial model parameters were derived from published literature and QUANTEC recommendations, [8, 9, 14–17] followed by institutional calibration. Final parameter values used in this study were: parotid (TD50 = 34.1 Gy, *m* = 0.11, *n* = 1.0), larynx (TD50 = 43.6 Gy, *m* = 0.16, *n* = 0.45) and oral cavity (TD50 = 48.5 Gy, *m* = 0.18, *n* = 1.0).

### ML implementation

Given the limited number of Grade ≥2 toxicity events per endpoint (3–5), ML analyses were undertaken for exploratory, hypothesis-generating purposes rather than for clinical model development or deployment. The available event counts are substantially below commonly recommended EPV thresholds for stable multivariable prediction modeling, [41, 42] and all ML results should therefore be interpreted as illustrative of relative trends within this dataset.

Feature engineering*:* A total of seventeen DVH–derived features were extracted for each organ, including mean dose, maximum dose, minimum dose, median dose, V10–V70 (volumes receiving ≥10–70 Gy in 10-Gy increments) and D2cc (minimum dose to the hottest 2 cm³). In addition, three clinical variables (age, sex and concurrent chemotherapy) were included, resulting in a total of 20 candidate predictors per endpoint.

Model architecture: ANN models consisted of a single hidden layer with ten nodes and ReLU activation, trained using binary cross-entropy loss and the Adam optimiser (learning rate 0.001), with a maximum of 100 epochs and early stopping (patience = 10 epochs). Extreme gradient boosting (XGBoost) models employed an ensemble of 100 decision trees with a maximum depth of 3, learning rate of 0.1 and L2 regularisation (*λ* = 1.0) [46].

Class imbalance handling: To address class imbalance, the Synthetic Minority Over-sampling Technique (SMOTE) [47] was applied exclusively to the training data, generating synthetic minority-class samples to achieve an approximate 1:2 class ratio (original ratios ranged from 1:10 to 1:16). This approach was applied conservatively, recognising that with very small minority-class sizes (*n* = 3–5), synthetic samples may incompletely reflect the underlying data distribution [48].

Hyperparameter optimisation: Hyperparameter tuning was performed using grid search with five-fold cross-validation on the training set. Owing to the low number of events, individual folds frequently contained zero or one event, limiting the stability of cross-validated optimisation and reinforcing the exploratory nature of these analyses [49].

### Statistical analysis

Performance metrics: Model discrimination was assessed using the area under the receiver operating characteristic curve (AUC), with 95% confidence intervals estimated using DeLong’s method for correlated ROC curves [50]. Given the very low event counts per endpoint (*n* = 3–5), Brier scores were considered unreliable for calibration assessment and are therefore not reported; this limitation is explicitly acknowledged. Accuracy is reported for completeness; however, given the binary endpoints and marked class imbalance, it primarily reflects the prediction of the majority class and was not used as a primary performance metric [51].

Correlation analysis: Because NTCP outputs are continuous probability estimates and toxicity grades are ordinal categorical variables, associations were evaluated using Spearman’s rank correlation coefficient (*ρ*) rather than Pearson’s correlation or linear regression. Squared Spearman correlations (*ρ*²) are reported to indicate the proportion of variance in toxicity grade rankings explained by NTCP-based rankings. This approach is more appropriate than linear regression–based *R*², which assumes continuous, normally distributed outcomes [52].

Model comparison: Differences in AUC between models were assessed using DeLong’s test for correlated ROC curves [50]. To account for multiple comparisons across endpoints and model pairs, the Bonferroni correction was applied (adjusted significance threshold *α* = 0.05/9 = 0.0056). Results were interpreted in the context of a limited sample size and event counts [53].

Computational agreement: Agreement between NTCP values generated by RBMODELv1 and the independent Python implementation was evaluated using Bland–Altman analysis [54]. Mean bias and 95% limits of agreement were calculated as bias ± 1.96 × SD of the differences. Acceptable agreement was predefined as a mean bias <3% and 95% limits of agreement within ±5%.

Confidence intervals: Bootstrap resampling (1,000 iterations, stratified by outcome) was used to generate additional 95% confidence intervals for AUC estimates. Given the low number of events per endpoint (3–5), bootstrap-based estimates may exhibit increased variability [55]. Accordingly, both DeLong-based and bootstrap confidence intervals are reported for completeness, with results interpreted cautiously.

Software: Statistical analyses were performed using R version 4.2.0 (packages: *pROC*, *caret*, *SMOTE*), Python 3.9 (*scikit-learn*, *XGBoost*), MATLAB R2021a (RBMODELv1) and SPSS version 26.

All statistical tests were two-tailed. In light of the limited sample size and class imbalance, emphasis was placed on effect sizes and confidence intervals rather than sole reliance on *p*-values, recognising that statistical significance does not necessarily imply clinical relevance or adequate statistical power [56, 57].

**Figure 1.**
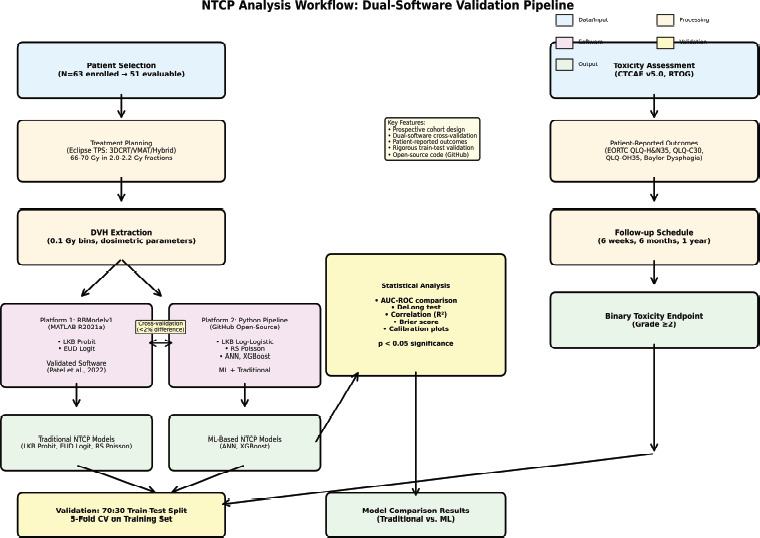
Study flowchart and computational framework.

The schematic diagram of the computational framework and workflow is shown in Figure 1.

Schematic overview of the dual computational pipeline used for NTCP modeling. The workflow shows: (A) Patient selection and DVH extraction, (B) Dual-pipeline NTCP calculation (RBMODV1(i.e., RBMODELv1) in MATLAB and Python implementation), (C) Traditional radiobiological modeling (LKB, EUD, RS), (D) Exploratory ML analysis (ANN, XGBoost with SMOTE) and (E) Statistical validation including Bland–Altman analysis for computational agreement and DeLong test for model comparison.

## Results

### Patient cohort and toxicity events

Patient and treatment characteristics are summarised in Table 1. The cohort was representative of a typical head and neck cancer population, with a median age of 56 years (range, 28–74), a predominance of male patients (66.7%), a high proportion of advanced-stage disease (70.6% Stage III–IV) and a mix of treatment techniques (3DCRT 45%, VMAT 55%). Grade ≥ 2 toxicity events were infrequent, with xerostomia observed in three patients (5.9%), dysphagia in five patients (9.8%) and mucositis in four patients (7.8%). While these low event rates are consistent with contemporary radiotherapy techniques and adherence to established dose constraints, they resulted in pronounced class imbalance across endpoints, with event-to-non-event ratios ranging from approximately 1:9 to 1:16. This imbalance limits the statistical power available for model discrimination analyses, particularly for multivariable and ML approaches [58]. Consistent with this, post-hoc power estimation indicated that the available sample size provided approximately 30%–40% power to detect an AUC difference of 0.15 at a two-sided *α* = 0.05 [40]

### DVH characteristics

Mean organ doses across the cohort were 42.8 ± 14.8 Gy for the parotid glands, 48.1 ± 11.9 Gy for the larynx and 48.7 ± 12.0 Gy for the oral cavity. Patients who developed Grade ≥ 2 toxicities generally received higher mean organ doses compared with those without such events, although differences did not consistently reach statistical significance. Specifically, mean parotid dose was 53.5 Gy versus 42.1 Gy (*p* = 0.204), mean laryngeal dose was 51.7 Gy versus 47.7 Gy (*p* = 0.527) and mean oral cavity dose was 60.8 Gy versus 47.7 Gy (*p* = 0.030) for patients with and without Grade ≥ 2 toxicity, respectively. These comparisons should be interpreted cautiously, given the limited number of events per endpoint. Only six patients (11.8%) achieved the QUANTEC-recommended constraint of a mean parotid dose ≤ 25 Gy, [6] reflecting the challenge of meeting stringent dose–volume objectives in routine clinical practice for locally advanced head and neck cancers.

3DCRT, three-dimensional conformal radiotherapy; VMAT, volumetric modulated arc therapy. Grade ≥2 toxicity defined per CTCAE v5.0 criteria

### Dual-pipeline computational validation

**Figure 2.**
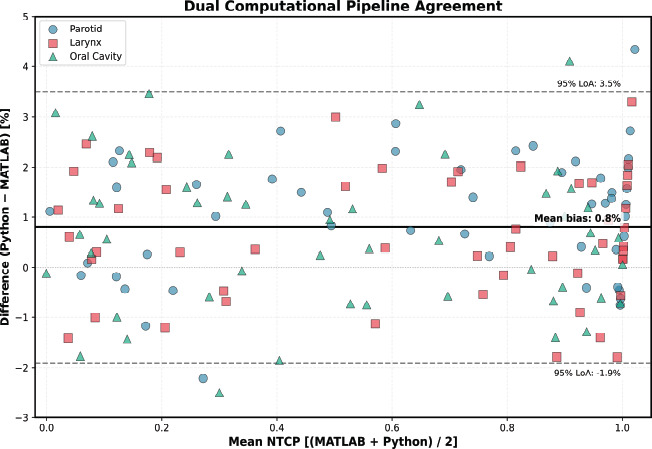
Bland-Altman analysis for computational validation.

Bland–Altman analysis demonstrated excellent agreement between the MATLAB-based and Python-based pipelines across all organs for the LKB model (Figure 2). The mean bias was 0.8%, with 95% limits of agreement ranging from −1.9% to +3.5%, meeting the predefined acceptability criteria (mean bias <3% and limits within ±5%). The maximum observed individual deviation was 2.4% and occurred at higher NTCP values (>80%), where small absolute differences result in larger relative percentage differences. Separate Bland–Altman analyses for the EUD and RS model outputs yielded comparable agreement bounds (mean bias <1.0%, 95% LoA within ±4.0% for all evaluated models); however, detailed panels are not presented as LKB served as the primary validation model for this study. Overall, these findings indicate that, when identical mathematical formulations and numerical conventions are applied, NTCP calculations are reproducible and computationally consistent across software platforms.

Bland–Altman plots demonstrating agreement between MATLAB-based RBMODELv1 and independent Python pipeline NTCP calculations for the LKB model across all three organs: (A) Parotid LKB model, (B) Larynx LKB model, (C) Oral cavity LKB model. *X*-axis shows mean NTCP between two implementations; *Y*-axis shows difference (Python - MATLAB) as percentage. Solid horizontal line indicates mean bias (0.8%); dashed lines show 95% limits of agreement (−1.9% to +3.5%). Agreement meets pre-defined acceptability criteria (bias <3%, limits within ±5%), confirming computational reproducibility. Maximum deviation of 2.4% occurred at high NTCP values (>80%). Detailed Bland–Altman panels for EUD and RS model outputs are not separately shown; EUD and RS agreement was confirmed to fall within comparable acceptability bounds (mean bias <1.0%, 95% LoA within ±4.0%).

### Traditional NTCP model performance

Benchmarking results for traditional NTCP models are summarised in Table 2. The LKB probit model demonstrated the highest discrimination across all three endpoints, with AUC values of 0.89 for parotid toxicity, 0.88 for laryngeal toxicity and 0.91 for oral cavity toxicity, although confidence intervals were wide owing to the limited number of events. The EUD-based model achieved comparable discrimination for dysphagia (AUC 0.83) and xerostomia (AUC 0.82), but showed lower performance for mucositis (AUC 0.64). The RS model demonstrated lower discrimination for parotid toxicity (AUC 0.59, 95% CI 0.46–0.72), consistent with the known limitations of serial-organ modeling when applied to predominantly parallel organ architectures such as the parotid glands.

**Figure 3.**
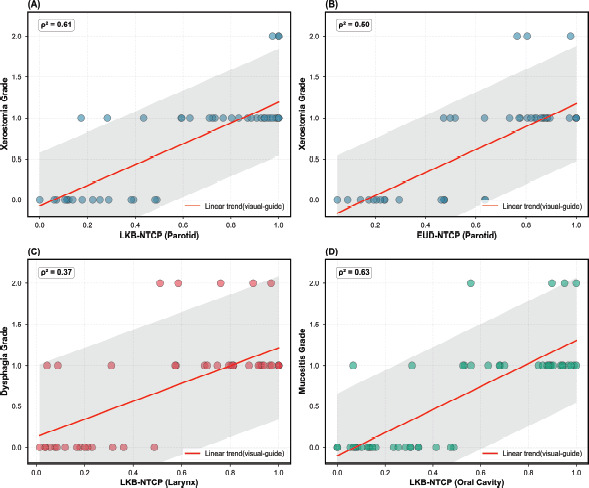
Correlation between NTCP and observed toxicity grades.

Rank-order associations between calculated NTCP values and observed toxicity grades were moderate to strong across endpoints (*ρ*² = 0.37–0.63, all *p* < 0.001), indicating robust relative risk stratification despite potential uncertainty in absolute probability calibration (Figure 3). Reported accuracy values (85.0%–94.1%) largely reflected correct classification of the majority class (absence of Grade ≥ 2 toxicity) and were therefore not interpreted as indicators of clinical utility in the context of severe class imbalance [51].

**Figure 4.**
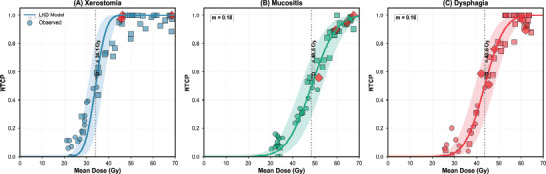
Institution-specific dose-response curves.

Institution-specific parameter estimation revealed systematic differences compared with QUANTEC-recommended values. In particular, the parotid TD50 was estimated at 34.1 Gy, compared with 39.0 Gy reported in QUANTEC, with a corresponding slope parameter m of 0.11 versus 0.18 as shown in the institute-specific dose-response curves (Figure 4). The detailed comparison of parameters is given in Table 3. These differences may reflect a combination of population-specific factors, differences in toxicity assessment methodology and contemporary treatment practices, including a higher proportion of VMAT use in the present cohort (55%) relative to earlier QUANTEC-era studies [1, 15, 59–62].

Scatter plots with regression lines showing correlation between calculated NTCP and observed toxicity grades (0, 1, 2). (A) Xerostomia vs. LKB-NTCP for parotid (*ρ*² = 0.61, *ρ* = 0.78, *p* < 0.001); (B) Xerostomia versus EUD-NTCP for parotid (*ρ*² = 0.50, *ρ* = 0.71, *p* < 0.001); (C) Dysphagia versus LKB-NTCP for larynx (*ρ*² = 0.37, *ρ* = 0.61, *p* < 0.001); (D) Mucositis versus LKB-NTCP for oral cavity (*ρ*² = 0.63, *ρ* = 0.79, *p* < 0.001). Solid lines represent ordinary least-squares (OLS) linear trend lines shown for visual reference only; statistical association was quantified by Spearman’s rank correlation coefficient (*ρ*). Dashed lines indicate 95% confidence bands of the OLS trend line. Strong rank-order correlations indicate robust relative risk stratification despite imperfect absolute probability calibration.

Sigmoid dose-response curves fitted with institution-specific LKB parameters. (A) Parotid xerostomia: TD50 = 34.1 Gy, *m* = 0.11, *n* = 1.0 (versus QUANTEC TD50 = 39.0 Gy); (B) Oral cavity mucositis: TD50 = 48.5 Gy, *m* = 0.18, *n* = 1.0 (versus QUANTEC TD50 = 50.0 Gy); (C) Larynx dysphagia: TD50 = 43.6 Gy, *m* = 0.16, *n* = 0.45 (versus QUANTEC TD50 = 46.0 Gy). Observed data points shown as circles (Grade 0), squares (Grade 1), and rhombus (Grade 2). Systematically lower TD50 values reflect population-specific factors and methodological differences from QUANTEC cohorts.

All correlations *p* < 0.001. **Accuracy values** (85.0%–94.1%) primarily reflect correct prediction of the majority class (Grade 0–1 toxicity) and should NOT be interpreted as indicators of clinical utility given severe class imbalance (events: non-events ratios approximately 1:9 to 1:16, consistent with Table 1 event counts). **AUC confidence intervals** are wide due to limited events (*n* = 3–5), indicating substantial statistical uncertainty in discrimination estimates. ***ρ²* values** represent squared Spearman rank correlations, NOT linear regression *R*², reflecting the proportion of variance in toxicity grade rankings explained by NTCP rankings. This is more appropriate for ordinal outcomes. **RS model’s poor performance** for parotid (AUC 0.59) likely reflects architectural mismatch – applying a serial-organ model to an inherently parallel structure. The RS model was applied only to the parotid gland for two reasons: (1) historical precedent – QUANTEC parameters [15] enabled direct comparison in Table 3; and (2) hypothesis testing – the poor performance empirically confirms that serial models are unsuitable for parallel organs. The RS model was not evaluated for the larynx (mixed architecture, no established parameters) or oral cavity (complex mucosa, serial formulation theoretically inappropriate). This selective application is a transparent limitation: parotid RS results serve primarily as historical reference and architectural validation, not as a clinically validated model. **Performance metrics** based on 70:30 train-test split with stratification. Cross-validation metrics not reported due to unreliability with <10 events per fold. **Statistical power** is inadequate (30%–40%) to detect clinically meaningful AUC differences of 0.15 at *α* = 0.05.

**Notes:** TD_50_, dose causing 50% complication probability; *m*, slope parameter; *n*, volume effect parameter; a, gEUD volume-effect parameter (negative = parallel, positive = serial); D_50_ (RS), dose causing 50% response in the RS model; *k*, dose–response steepness; *s*, seriality parameter (0 = purely parallel, 1 = purely serial); PRO, patient-reported outcomes; VMAT, volumetric modulated arc therapy

Systematically lower TD_50_ values in this study likely reflect: (1) genetic polymorphisms in South Asian populations affecting radiation sensitivity, (2) EORTC PRO instruments detecting toxicity earlier than LENT–SOMA physician ratings and (3) different spatial dose distributions with modern VMAT techniques.

RS model applicability: The RS model was applied only to the parotid gland for two reasons: (1) historical precedent – QUANTEC parameters [15] enabled direct comparison; and (2) hypothesis testing – the poor discrimination performance (AUC 0.59 in Table 2) empirically confirms that serial models are unsuitable for parallel organs. RS was not evaluated for larynx (mixed architecture, no established RS parameters) or oral cavity (complex mucosa, serial formulation theoretically inappropriate). These parameters are reported for completeness and transparency; their inclusion does not imply clinical validation of the RS model for parotid applications.

QUANTEC model parameters were taken from organ-specific QUANTEC publications: parotid glands [15], larynx/pharynx [16] and oral mucosa based on pooled QUANTEC consensus summaries [6, 14].

### Exploratory ML analysis

**Figure 5.**
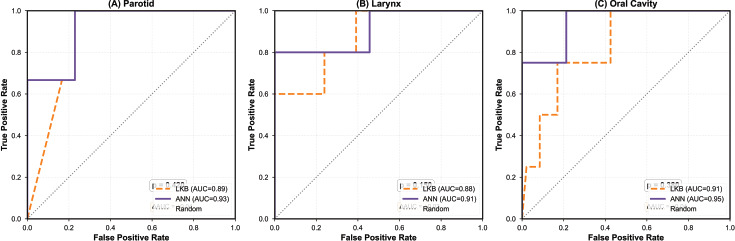
Exploratory ROC curve comparison: traditional versus ML models.

Exploratory ML analyses were performed to examine whether data-driven approaches exhibit organ-dependent performance patterns when applied to the same dosimetric inputs used in traditional NTCP modeling. Given the limited number of Grade ≥ 2 toxicity events per endpoint in this cohort, these analyses were conducted for hypothesis-generating purposes rather than to support claims of clinical superiority. The small number of observed events (3–5 per endpoint) limits the statistical stability of multivariable models, and test-set performance is therefore sensitive to minor variations in event distribution [41, 42, 55]. In addition, although class-imbalance mitigation using SMOTE and internal cross-validation was applied exclusively within training data, very low event counts may restrict the representativeness of synthetic samples and internal validation estimates [48]. No external validation cohort was available, and statistical significance alone should not be interpreted as evidence of clinical utility or generalisability. Within these constraints, Table 4 and Figure 5 summarise exploratory performance trends. On the independent hold-out test set, ANN models were compared against the best-performing traditional model for each organ (LKB for all three). For the parotid glands, ANN (AUC 0.93) showed a modest numerical improvement over LKB (AUC 0.89; ΔAUC = +0.04; *p* = 0.420). For the oral cavity, ANN (AUC 0.95) showed a similar modest advantage over LKB (AUC 0.91; ΔAUC = +0.04; *p* = 0.380). For the mixed-architecture larynx, performance differences were similarly small and non-significant (ANN AUC 0.91 versus LKB 0.88; ΔAUC = +0.03; *p* = 0.150).

These findings support a hypothesis warranting further investigation in adequately powered, multi-institutional cohorts: ML approaches may offer relative advantages for parallel-architecture organs characterised by complex spatial dose distributions and functional subunit redundancy, whereas mechanistic radiobiological models may remain sufficient for organs with more straightforward dose–response relationships [42, 63].

ROC curves comparing traditional models and exploratory ML on hold-out test sets, using the best-performing traditional model (LKB) for each organ as a comparator. (A) Parotid xerostomia: ANN (AUC = 0.93, 95% CI 0.79–1.00) versus best traditional LKB model (AUC = 0.89, 95% CI 0.82–0.96), *p* = 0.420 by DeLong test; (B) Larynx dysphagia: ANN (AUC = 0.91, 95% CI 0.82–1.00) versus best traditional LKB model (AUC = 0.88, 95% CI 0.79–0.95), *p* = 0.150; (C) Oral cavity mucositis: ANN (AUC = 0.95, 95% CI 0.87–1.00) versus best traditional LKB model (AUC = 0.91, 95% CI 0.85–0.97), *p* = 0.380. *Note:* This is an exploratory ML model performance across organs. Results are hypothesis-generating only due to limited event counts (3–5 per endpoint). No statistically significant differences were observed between ANN and the best traditional model (LKB) for any organ. Statistical significance does not indicate clinical utility or generalisability.

Extreme gradient boosting (XGBoost) models showed lower generalisation performance compared with ANN across all endpoints, with evidence of overfitting despite regularisation (training AUC 0.98–1.00; test AUC 0.67–0.82). This overfitting pattern constitutes a methodologically important negative finding that further reinforces the cautionary narrative regarding ML in low-event settings. To maintain clarity and focus on the primary comparative patterns observed, detailed XGBoost results are provided as Supplementary Table S1.

No results were statistically significant (*p* < 0.05, DeLong test). †DeLong test for correlated ROC curves. Best traditional model for all organs: LKB (the highest performing traditional model per Table 2). ANN, artificial neural network; AUC, area under the curve; ΔAUC, difference in AUC (ANN minus best traditional model: LKB); LKB, Lyman–Kutcher–Burman

Notes: ***Severe class imbalance:*** Test sets contained only 1–2 positive events, rendering AUC estimates statistically unstable and confidence intervals unreliable. ***Violated sample size requirements:*** With 3–5 events total, the ML analysis violates established EPV guidelines (≥10 events per predictor). The 17–20 feature models represent extreme overfitting risk. ***No external validation:*** All results from the single-institution dataset; generalisability unknown. External validation in independent cohorts is absolutely essential. ***SMOTE limitations:*** Synthetic oversampling with *n* = 3–5 training events creates artificial data that may not represent the true minority class distribution. This is a fundamental methodological concern. ***Cross-validation unreliability:*** With 3–5 events, cross-validation folds contained 0–1 events, rendering hyperparameter optimisation unreliable. ***Statistical instability:*** DeLong test *p*-values are questionable with extreme class imbalance. Bootstrap confidence intervals similarly unreliable. Statistical Significance ≠ Clinical Utility: *p* < 0.05 does NOT indicate clinical readiness, generalisability or superiority over traditional models. ***Appropriate interpretation:*** These exploratory findings suggest a hypothesis – ML may offer advantages for parallel-architecture organs – that requires validation in multi-institutional cohorts with ≥50 events per endpoint before any clinical consideration.

### Feature importance analysis

**Figure 6.**
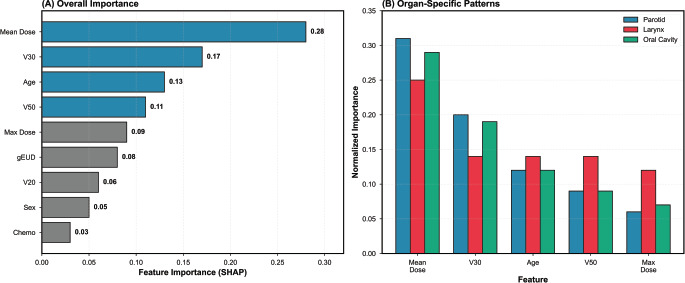
Feature importance from SHAP analysis.

Feature importance was explored using SHapley Additive exPlanations (SHAP) analysis applied to the ANN models. Across all organs, mean dose emerged as the most influential predictor (mean SHAP value 0.28), followed by intermediate dose–volume parameters such as V30 (0.17) and patient age (0.13) (Figure 6). DVH metrics reflecting mid-to-high dose regions (e.g., V30 and V50) consistently ranked higher than low-dose volume parameters.

These patterns are consistent with established radiobiological understanding and prior NTCP literature, which emphasise the relevance of mean dose and intermediate dose–volume exposure in normal tissue toxicity prediction. Given the exploratory nature of the ML analyses and limited event counts, feature importance rankings should be interpreted as indicative of relative trends rather than definitive evidence of causal relationships.

Bar plot showing mean SHAP values across all three organ models, indicating the relative importance of features in ANN predictions. Top predictors: mean dose (0.28), V30 (0.17), patient age (0.13), V50 (0.11), maximum dose (0.09), gEUD (0.08), V20 (0.06), sex (0.05), concurrent chemotherapy (0.03). DVH metrics capturing mid-to-high dose regions (V30, V50) consistently ranked higher than low-dose volumes, supporting QUANTEC emphasis on mean dose and intermediate dose-volume parameters for toxicity prediction. However, given severe class imbalance (3–5 events), feature importance estimates should be interpreted as exploratory observations only.

### Sensitivity analysis with grade ≥1 endpoint

To evaluate the stability of NTCP model performance under conditions of higher event frequency and to explore the impact of severe class imbalance observed in the primary analysis, a sensitivity analysis was performed using a lower toxicity threshold (Grade ≥1). This redefinition resulted in substantially higher event rates across endpoints: xerostomia (*n* = 41, 80.4%), dysphagia (*n* = 38, 74.5%) and mucositis (*n* = 39, 76.5%). Using this alternative endpoint, traditional NTCP models demonstrated discrimination patterns broadly consistent with the primary analysis. LKB model performance remained moderate, with AUC values of 0.82 (95% CI: 0.72–0.92) for parotid toxicity, 0.79 (95% CI: 0.67–0.91) for laryngeal toxicity and 0.84 (95% CI: 0.74–0.94) for oral cavity toxicity. The preservation of rank-order discrimination across endpoints suggests that the underlying dose–response relationship is detectable even when less severe toxicity definitions are applied. However, Grade ≥1 toxicity generally corresponds to mild or transient symptoms that do not require clinical intervention, limiting the direct clinical relevance of this endpoint compared with Grade ≥2 toxicity. Accordingly, this sensitivity analysis should be interpreted as supportive evidence for biological plausibility and model stability rather than as a substitute for the primary, clinically meaningful endpoint. Together with the primary analysis, these findings indicate that while Grade ≥2 toxicity remains the appropriate clinical endpoint, lower-grade toxicity analyses can provide complementary insight into dose–response behaviour in small cohorts where event rates are limited.

## Discussion

This institutional validation study establishes a reproducible computational framework for NTCP modeling in head and neck radiotherapy through dual-pipeline verification and derives population-specific model parameters for an Indian patient cohort. Traditional radiobiological models demonstrated acceptable discrimination following local calibration; however, the low number of Grade ≥ 2 toxicity events (3–5 per endpoint) imposes important constraints on statistical stability and precludes definitive conclusions regarding the added value of ML–based augmentation. Accordingly, all ML findings are interpreted within a hypothesis-generating framework.

### Computational validation and reproducibility

A key contribution of this work is the demonstration that independent computational implementations – MATLAB-based RBMODELv1 and a custom Python-based pipeline – produce highly concordant NTCP estimates, with a mean bias of 0.8% and narrow 95% limits of agreement (−1.9% to +3.5%). This finding directly addresses long-standing concerns regarding inter-software variability that have limited broader clinical adoption of NTCP models [64, 65]. Prior reports have documented discrepancies of up to 10%–15% in NTCP or EUD calculations across platforms, attributable to differences in dose–volume interpolation, numerical integration schemes and rounding conventions [28, 30, 65]. Our results indicate that when identical mathematical formulations and numerical conventions are implemented with sufficient precision, computational consistency is achievable across software environments. Such reproducibility is essential for (1) independent validation of published NTCP models, (2) quality assurance of clinical decision-support tools and (3) multi-institutional collaborative studies requiring standardised calculations across participating centers [66].

RBMODELv1, developed at our institution, provides a validated, transparent platform for NTCP estimation using established radiobiological models [45]. Cross-verification against an independent Python implementation based on widely used scientific computing libraries (NumPy, SciPy) further enhances transparency and auditability, which are increasingly important for regulatory-compliant analyses and prospective clinical studies. On this basis, we recommend the adoption of similar dual-validation strategies by institutions seeking to implement NTCP-based treatment plan evaluation in routine practice.

## Population-specific parameter calibration

Institution-specific LKB parameter estimates differed systematically from QUANTEC-recommended values across organs, with lower TD50 estimates observed for the parotid glands (34.1 Gy versus QUANTEC 39.0 Gy), oral cavity (48.5 Gy versus 50.0 Gy) and larynx (43.6 Gy versus 46.0 Gy). Differences of approximately 4–5 Gy are potentially clinically relevant and likely reflect the combined influence of population-specific, methodological and treatment-related factors rather than a single causal mechanism.

One contributing factor may be inter-population biological variability. Genetic polymorphisms in DNA damage response and repair pathways have been reported at higher prevalence in South Asian populations and have been associated with increased radiation sensitivity in prior studies [18, 19, 67]. In addition, methodological differences in toxicity assessment may influence estimated dose–response parameters. In the present study, toxicity grading was informed by patient-reported outcomes using EORTC quality-of-life instruments, whereas QUANTEC parameters were largely derived from physician-reported LENT–SOMA scales. Patient-reported measures have been shown to capture symptom onset earlier and at lower dose levels, which may contribute to lower apparent TD50 estimates [68]. Differences in treatment technique and dose distribution also warrant consideration. A higher proportion of patients in the present cohort were treated with VMAT (55%) compared with cohorts contributing to QUANTEC analyses (approximately 30%), potentially resulting in altered spatial dose patterns and volume-effect relationships that are not fully captured by historical parameters [69].

Consistent with these observations, population-specific NTCP parameter variation has been reported in other Asian cohorts. Single-institution Japanese IMRT studies have demonstrated parotid TD50 estimates lower than those commonly reported in Western series [70]. Similarly, Chinese nasopharyngeal cancer cohorts, which model *delivered* rather than planning dose, demonstrate dose–response behavior that differs from older Western datasets [71], with several studies from Chinese populations reporting steeper dose–response slopes than QUANTEC-derived estimates [72]. Systematic reviews further confirm substantial inter-study variability in NTCP model parameters across cohorts, including Asian NPC populations [73].

Collectively, these findings underscore the dependence of NTCP model parameters on endpoint definition, cohort characteristics and treatment paradigm and they reinforce QUANTEC’s recommendation for institutional validation and local calibration of dose–response parameters rather than universal adoption of published values [15, 25, 61, 70, 71, 74].

### Performance of traditional NTCP models

Traditional radiobiological models demonstrated moderate to strong rank-order associations with observed toxicity grades (*ρ*² = 0.37–0.63), indicating effective relative risk stratification despite uncertainty in absolute probability calibration. This observation is consistent with systematic reviews showing that NTCP models generally perform better at distinguishing higher-risk from lower-risk patients than at providing precise absolute risk estimates without local calibration [7, 75, 76]. In the context of treatment planning optimisation and comparative plan evaluation, such relative risk ranking may be sufficient to support clinical decision-making [77]. In contrast, applications requiring individualised risk communication or patient selection for clinical trials necessitate calibrated absolute probability estimates [78]. Among the evaluated formulations, the LKB model demonstrated consistently higher discrimination than the EUD- and RS-based approaches across endpoints. This finding may reflect the greater flexibility afforded by the LKB model’s parameterisation, which separately accounts for dose–response steepness (*m*), volume dependence (*n*) and overall sensitivity (TD50). In comparison, the lower performance of the RS model for parotid toxicity (AUC 0.59) highlights the importance of matching model assumptions to underlying organ architecture, as application of serial-organ formulations to predominantly parallel structures may reduce predictive performance [12, 13].

### ML: promises and pitfalls in small datasets

Our exploratory ML analysis was designed to generate hypotheses rather than to meet standards for clinical model validation. The limited number of Grade ≥2 toxicity events (3–5 per endpoint) constrains the stability of multivariable prediction and necessitates cautious interpretation of model performance [41, 42, 58, 79]. Established guidance based on EPV considerations suggests that substantially larger event counts are required for reliable multivariable ML, and model performance estimates derived from small event numbers are known to be statistically unstable [41, 42]. Simulation studies have further demonstrated that ML models trained on very small numbers of events can exhibit wide variability in discrimination metrics, including large fluctuations in AUC estimates across resampling procedures [55, 80]. Further, in this study, the extreme gradient boosting (XGBoost) results, with near-perfect training performance but substantially lower test set discrimination, exemplify the high risk of overfitting when complex, flexible algorithms are applied to small datasets with limited events. This negative finding underscores that ML approaches are not inherently superior and require rigorous validation with adequate sample sizes before any clinical consideration.

Within these limitations, the observed numerical differences between ML and traditional models are best interpreted as hypothesis-generating. When comparing ANN against the best-performing traditional model (LKB) for each organ, differences were modest across all endpoints: parotid glands (ΔAUC +0.04), oral cavity (ΔAUC +0.04) and mixed-architecture larynx (ΔAUC +0.03), no statistically significant advantage for ML was observed for any organ architecture. Parallel organs are characterised by functional subunit redundancy, spatial dose heterogeneity and compensatory mechanisms that may introduce non-linear dose–response relationships not fully captured by parametric radiobiological models [81]. In principle, ML approaches, which are not constrained by predefined functional forms, may be capable of modeling such complex interactions [31, 82, 83]. However, confirmation of this hypothesis requires substantially larger cohorts, with recommended event counts of at least 50 per endpoint, preferably through multi-institutional data pooling [63, 66].

Recent large-scale studies provide context. Dean *et al* [84] (*n* = 130, 45 events) found ML advantages for oral mucositis prediction (AUC 0.82 versus 0.71 traditional). El Naqa *et al* [31] used multivariable ML approaches for radiotherapy outcomes modeling, noting organ-architecture-dependent differences, supporting the organ-architecture hypothesis. However, a systematic review of ML-based toxicity prediction in radiotherapy concluded that consistent superiority of ML over conventional models remains undemonstrated, primarily due to inadequate external validation, heterogeneous study designs and small sample sizes across the published literature [38].

### Clinical implementation barriers

Despite the acceptable performance of traditional radiobiological models in this and prior studies, several practical factors continue to limit the routine clinical adoption of NTCP-based evaluation. These include the computational complexity of model implementation, which often requires specialised software or in-house expertise; the absence of universally accepted parameter sets, necessitating institutional validation and recalibration; limited prospective evidence demonstrating that NTCP-optimised treatment planning improves patient outcomes compared with standard DVH-constraint-based approaches [64, 85] and the lack of widespread integration of NTCP models into regulatory-approved commercial treatment planning systems. Collectively, these considerations help explain why, despite more than four decades of development since Lyman’s foundational work [8], NTCP models have remained more commonly used in research and planning studies than in routine clinical workflows.

### Limitations and future directions

In addition to the low incidence of Grade ≥2 toxicity events, several methodological considerations should be acknowledged when interpreting these findings. The retrospective study design may introduce selection and information biases, and the single-institution setting limits generalisability to other populations and treatment environments. Follow-up duration was heterogeneous (median 8 months, range 3–24 months), which may reduce sensitivity for detecting late-onset toxicities. Baseline patient-reported outcome measures were not uniformly available, limiting the ability to quantify treatment-related changes over time. Furthermore, the absence of validation in independent external cohorts constrains assessment of model transportability, and the use of binary toxicity endpoints, while clinically practical, does not fully exploit time-to-event information that could better accommodate censoring and temporal patterns of toxicity [86]. The selection of Grade ≥2 as the binary toxicity threshold was guided by clinical relevance, as Grade ≥2 events typically represent functionally significant complications warranting clinical intervention; however, this threshold substantially reduced the number of positive events per endpoint (3–5 events), thereby limiting statistical power. The sensitivity analysis at Grade ≥1 yielded consistent directional findings with improved event counts, providing supporting biological plausibility for the observed dose–response relationships while acknowledging that Grade 1 events may include clinically insignificant toxicities. Future investigations should aim to address these considerations through coordinated multi-institutional data aggregation with sufficient event counts per endpoint to support robust model development and validation [66]. Integration of genetic and molecular biomarkers, such as single-nucleotide polymorphisms in DNA repair pathways, alongside dosimetric features, may enhance individualised toxicity prediction [87]. Spatially resolved dose analyses using voxel-based or regional approaches could further refine structure–function relationships [88]. Prospective studies comparing NTCP-guided treatment planning with conventional DVH-based strategies are needed to determine clinical benefit, and federated learning frameworks offer a promising pathway for collaborative model training across institutions while preserving data privacy [89]. Furthermore, variation in organ-at-risk dose and biologically effective dose across different α/β values for conventional, moderate and ultra-hypofractionated treatment regimens represents an important consideration when evaluating the transferability of NTCP model parameters derived from standard fractionation cohorts, such as the present one, to emerging hypofractionation schedules [90].

An additional methodological consideration concerns the selective application of the RS model – applied only to the parotid gland and not to larynx or oral cavity. This deliberate choice was guided by historical precedent (QUANTEC parameters available for comparison) and the opportunity to empirically confirm that serial models underperform for parallel organs (AUC 0.59), reinforcing the organ-architecture theme of this study. The absence of established RS parameters for larynx/dysphagia and oral cavity/mucositis endpoints precluded their evaluation. Readers should therefore interpret the parotid RS results primarily as a historical reference and hypothesis test rather than a clinically validated model. This asymmetry is inherent to our study design and discussed in Table 2 notes.

## Conclusion

This study establishes a reproducible dual-pipeline framework for NTCP modeling and derives population-specific parameters for an Indian head and neck cancer cohort. Following local calibration, traditional radiobiological models demonstrated robust toxicity risk stratification, with the LKB model achieving the highest discrimination (AUC 0.88–0.91) and institution-specific parameters differing from QUANTEC values. Exploratory ML analyses suggest a potential organ-architecture–dependent performance pattern, warranting validation in adequately powered multi-institutional cohorts. The validated framework provides a foundation for future prospective studies assessing the clinical value of NTCP-guided treatment planning.

## List of abbreviations

3DCRT, three-dimensional conformal radiotherapy; AJCC, American Joint Committee on Cancer; ANN, artificial neural network; AUC, area under the curve; CI, confidence interval; CT, computed tomography; CTCAE, Common Terminology Criteria for Adverse Events; DVH, dose-volume histogram; EORTC, European Organisation for Research and Treatment of Cancer; EPV, events per variable; EUD, equivalent uniform dose; gEUD, generalised equivalent uniform dose; LKB, Lyman–Kutcher–Burman; ML, machine learning; NTCP, normal tissue complication probability; OAR, organ at risk; QUANTEC, Quantitative Analyses of Normal Tissue Effects in the Clinic; RBMODv1, RBMODELv1(Radiobiological Model Version 1); ROC, receiver operating characteristic; RS, relative seriality; SMOTE, Synthetic Minority Over-sampling Technique; VMAT, volumetric modulated arc therapy; XGBoost, extreme gradient boosting

## Conflicts of interest

The authors declare no conflicts of interest.

## Funding

This research did not receive any specific grant from funding agencies in the public, commercial or not-for-profit sectors.

## Ethical approval

This study involved retrospective analysis of de-identified clinical and dosimetric data and had no impact on patient management. In accordance with institutional policy, formal ethical approval was exempted by the ethics committee (Banaras Hindu University).

## Use of AI-assisted tools

AI-assisted tools (Claude, Anthropic) were used for language editing and manuscript formatting during the revision process. All scientific content, data analyses, interpretations and conclusions were performed solely by the listed authors, who take full responsibility for the accuracy and integrity of the work.

## Author contributions

KM: Conceptualisation, Methodology, Software, Formal Analysis, Data Curation, Writing – Original Draft, Visualisation. AM: Methodology, Validation, Writing – Review & Editing, Supervision. AV: Resources, Writing – Review & Editing, Supervision. GP: Conceptualisation, Methodology, Software, Validation, Writing – Review & Editing, Supervision, Project Administration. All authors reviewed and approved the final manuscript.

## Figures and Tables

**Table S1.** Detailed performance metrics for extreme gradient boosting (XGBoost) models.

Organ	Training AUC	Test AUC	Test accuracy (%)	Test precision	Test recall	Test F1-score
Parotid	0.99	0.71	88.2	0.33	0.50	0.40
Larynx	0.98	0.67	86.4	0.40	0.67	0.50
Oral cavity	1.00	0.82	85.0	0.25	0.50	0.33

**Notes:** *Severe overfitting evident:* Near-perfect training performance (AUC 0.98–1.00) with substantially lower test set discrimination (AUC 0.67–0.82) despite L2 regularisation (*λ* = 1.0) and limited tree depth (max_depth = 3). *Class imbalance impact:* Test sets contained only 1–2 positive events per organ, rendering precision, recall and F1-score estimates statistically unstable. *EPV violation:* With only 3–5 events per endpoint and 20 candidate predictors, XGBoost models grossly violate minimum EPV guidelines (≥10 events per predictor), making overfitting inevitable. *Interpretation:* These results serve as a methodologically important negative finding, demonstrating that complex machine learning algorithms require substantially larger event counts for reliable performance. The overfitting pattern reinforces the cautionary narrative regarding ML in low-event settings. *Comparison:* ANN models (Table 4) showed better generalisation (test AUC 0.91–0.95) than XGBoost, suggesting that simpler architectures may be preferable when events are limited

**Table 1.** Patient and treatment characteristics (N = 51).

Characteristic	Value
Age, median (range), years	56 (28–74)
Sex: Male/Female, *n* (%)	34 (66.7)/17 (33.3)
Primary site, *n* (%)	
Larynx	17 (33.3)
Tongue	14 (27.5)
Buccal mucosa	9 (17.6)
Others	11 (21.6)
Disease stage: I–II/III–IV, *n* (%)	15 (29.4)/36 (70.6)
Treatment technique, *n* (%)	
3DCRT	23 (45.1)
VMAT	28 (54.9)
Concurrent chemotherapy, *n* (%)	21 (41.2)
Total dose, mean ± SD, Gy	67.7 ± 1.2
Toxicity outcomes (Grade ≥2)	
Xerostomia, *n* (%)	3 (5.9)
Dysphagia, *n* (%)	5 (9.8)
Mucositis, *n* (%)	4 (7.8)
Mean organ doses, Gy	
Parotid (mean ± SD)	42.8 ± 14.8
Larynx (mean ± SD)	48.1 ± 11.9
Oral cavity (mean ± SD)	48.7 ± 12.0

**Table 2.** Traditional radiobiological model performance benchmarking.

Organ (Toxicity)	Model	AUC (95% CI)	Accuracy (%)	ρ²
Parotid (Xerostomia)				
	LKB Probit	0.89 (0.82–0.96)	94.1	0.61
	EUD Logit	0.82 (0.74–0.90)	88.2	0.50
	RS Poisson	0.59 (0.46–0.72)	85.0	0.47
Larynx (Dysphagia)				
	LKB Probit	0.88 (0.79–0.95)	90.9	0.37
	EUD Logit	0.83 (0.73–0.92)	86.4	0.37
Oral cavity (Mucositis)				
	LKB Probit	0.91 (0.85–0.97)	90.0	0.63
	EUD Logit	0.64 (0.52–0.76)	85.0	0.09

Note: AUC, area under the curve; CI, confidence interval; LKB, Lyman-Kutcher-Burman; EUD, equivalent uniform dose; RS, relative seriality; *ρ*², squared Spearman rank correlation

**Table 3.** Comparison of institution-specific NTCP model parameters with QUANTEC-recommended values.

Organ	Model	Parameter	This study	QUANTEC [Ref]	Diff.	Possible explanation
LKB model parameters						
Parotid	LKB	TD_50_ (Gy)	34.1	39.0 [15]	−4.9	Population genetics; PRO versus physician-rated toxicity
		m	0.11	0.18	−0.07	Steeper dose–response in this cohort
		n	1.0	1.0	0.0	—
Larynx	LKB	TD_50_ (Gy)	43.6	46.0 [16]	−2.4	Treatment technique differences (VMAT 55% versus ~30%)
		m	0.16	0.16	0.0	—
		n	0.45	0.45	0.0	—
Oral Cavity	LKB	TD_50_ (Gy)	48.5	50.0 [6,14]	−1.5	Similar to parotid; PRO detection earlier
		m	0.18	0.18	0.0	—
		n	1.0	1.0	0.0	—
gEUD volume-effect parameter						
Parotid	gEUD	a	−1	−1 [15]	0.0	Parallel architecture; consistent with QUANTEC classification
Larynx	gEUD	a	+3	+3 [16]	0.0	Mixed architecture (serial-dominant); consistent with QUANTEC
Oral Cavity	gEUD	a	−1	−1 [6,14]	0.0	Parallel architecture designation
RS model parameters (Parotid only)						
Parotid	RS	D_50_ (Gy)	34.1	39.9 [15]	−5.8	Same trend as LKB TD_50_; population-specific shift
		k	2.1	2.11	−0.01	—
		s	0.05	0.07	−0.02	Lower seriality reflects parallel organ nature

**Table 4.** Exploratory ML model performance comparison (hold-out test set).

Organ	Best traditional (AUC)	ANN (AUC)	ΔAUC	p-value†
Parotid	LKB: 0.89	0.93	+0.04	0.420
Larynx	LKB: 0.88	0.91	+0.03	0.150
Oral cavity	LKB: 0.91	0.95	+0.04	0.380
